# Cranial morphology of the tanystropheid *Macrocnemus bassanii* unveiled using synchrotron microtomography

**DOI:** 10.1038/s41598-020-68912-4

**Published:** 2020-07-24

**Authors:** Feiko Miedema, Stephan N. F. Spiekman, Vincent Fernandez, Jelle W. F. Reumer, Torsten M. Scheyer

**Affiliations:** 1 Palaeontological Institute and Museum, University of Zürich, Karl Schmid-Strasse 4, 8006 Zurich, Switzerland; 2 Faculty of Geosciences, Utrecht University, Princetonlaan 8a, 3584 CB Utrecht, The Netherlands; 3 Staatliches Museum für Naturkunde Am Löwentor, Rosenstein 1, 70191 Stuttgart, Germany; 4 European Synchrotron Radiation Facility, 71 avenue des Martyrs, 38000 Grenoble, France; 5 Natural History Museum, Cromwell Road, London, SW7 5BD UK; 6 Naturalis Biodiversity Center, P.O. Box 9517, 2300 RA Leiden, The Netherlands; 7 Natuurhistorisch Museum Rotterdam, Westzeedijk 345, 3015 AA Rotterdam, The Netherlands

**Keywords:** Evolution, Palaeontology, Herpetology

## Abstract

The genus *Macrocnemus* is a member of the Tanystropheidae, a clade of non-archosauriform archosauromorphs well known for their very characteristic, elongated cervical vertebrae. Articulated specimens are known from the Middle Triassic of Alpine Europe and China. Although multiple articulated specimens are known, description of the cranial morphology has proven challenging due to the crushed preservation of the specimens. Here we use synchrotron micro computed tomography to analyse the cranial morphology of a specimen of the type species *Macrocnemus bassanii* from the Besano Formation of Monte San Giorgio, Ticino, Switzerland. The skull is virtually complete and we identify and describe the braincase and palatal elements as well the atlas-axis complex for the first time. Moreover, we add to the knowledge of the morphology of the skull roof, rostrum and hemimandible, and reconstruct the cranium of *M. bassanii* in 3D using the rendered models of the elements. The circumorbital bones were found to be similar in morphology to those of the archosauromorphs *Prolacerta broomi* and *Protorosaurus speneri*. In addition, we confirm the palatine, vomer and pterygoid to be tooth-bearing palatal bones, but also observed heterodonty on the pterygoid and the palatine.

## Introduction

The genus *Macrocnemus* was described in the early twentieth century by Baron von Nopcsa based on a fragmentary specimen from Monte San Giorgio^1,2^. After subsequent expeditions to the Besano Formation, which crops out in the Monte San Giorgio area, resulted in the discovery of additional specimens, the taxon was more properly described^3^. Unfortunately, the holotype, stored in Milan, has since been lost as it was destroyed during the Second World War^4^. The phylogenetic affinities of *Macrocnemus* were initially unclear. Early researchers observed similarities in the shape of the cervical vertebrae with contemporaneous taxa and erected the taxon Protorosauria^5,6^. Notable members of this formerly recognised ‘superorder’ were *Tanystropheus*, *Protorosaurus*, *Macrocnemus* and *Prolacerta*, and all these taxa have elongated cervical vertebrae. The elongation of the cervical vertebrae is expressed most extremely in the genus *Tanystropheus,* of which the neck is three times the length of its trunk^7^. Researchers were unsure whether to place the clade closer to the Lepidosauria or to the Archosauromorpha (e.g.^7,8^). Currently, the members of the Protorosauria are all considered non-archosauriform archosauromorphs (e.g.^9–11^). However, the clade itself has been observed to be paraphyletic or even polyphyletic in recent phylogenetic analyses^9^ and references therein. Nonetheless, the recent analyses noted above have all recovered a monophyletic Tanystropheidae at the base of Archosauromorpha that includes *Macrocnemus* as well as *Tanystropheus*, *Amotosaurus*, *Langobardisaurus*, and *Tanytrachelos,* with *Jesairosaurus* often recovered as sister taxon to the clade.

There are currently three recognised species of *Macrocnemus*. The type species *M. bassanii* Nopcsa, 1930 ^1^ is known from the Besano Formation and Meride Limestone of Monte San Giorgio^3,8,12^ and found on both sides of the Italian-Swiss border. The species is known from roughly a dozen articulated or associated specimens, with cranial and postcranial elements. Its distinctive synapomorphies compared to other tanystropheids are the u-shaped suture between frontals and the parietals and the relatively long limb elements^9^. The other two species have been described more recently. *Macrocnemus fuyuanensis* Li, Zhao and Wang, 2007 ^13^, is known from the Middle Triassic of China. Both the holotype and referred specimens are (semi-) articulated and contain both cranial and postcranial elements^13,14^. This species is distinguished from other species of *Macrocnemus* based on limb bone ratios in which its humerus is significantly longer than its radius and its femur is relatively longer than its tibia-fibula in contrast to *M. bassanii*^13^. Recently, a specimen from the Besano Formation of Switzerland has been assigned to *Macrocnemus* aff. *fuyuanensis* based on the limb ratios and interclavicle morphology of the specimen, considered closer to that of the Chinese taxon than to that of *M. bassanii*^12^. The third species, *M. obristi* Fraser and Furrer, 2013 is known from the Middle Triassic of canton Grisons (= Graubünden), Switzerland^4^. Only posterior postcranial material is known, for both the type and a referred specimen. Like *M. fuyuanensis, M. obristi* was distinguished from the other two species based on limb ratios, in this case the ratio between the femur and the tibia-fibula length, whereby *M. obristi* had relatively longer tibiae-fibulae than *M. bassanii* and the holotype of *M. fuyuanensis*^4^. Similarly, *Tanystropheus longobardicus* might also have had a similar distribution^15^, and two species of *Tanystropheus* might also have co-occurred at Monte San Giorgio^14^. The close resemblance between these taxa known from opposite ends of the Tethys coastline, as well as the possible occurrence of the same species in both China and western Europe, sheds light on the diversification and dispersal of vertebrate taxa in the Middle Triassic, relatively shortly after the Permo-Triassic mass extinction^12–16^.

Despite the seemingly well preserved and articulated specimens, there has been some disagreement on the cranial morphology of *Macrocnemus*. Taphonomical factors, such as compression, caused all specimens to be extremely flattened. Cranial elements are often crushed or obscured by either matrix or bone. This has led to complications on the identification of elements, especially so in the posterior skull region and the palate. It caused uncertainty about the exact position and morphology of lateral and rostral elements^8,12,14,17^. This led to different interpretations on the morphology, especially of the referred specimen of *M. fuyuanensis*, whereby the authors of the original description suggested a morphology that is widely different from that of *M. bassanii* to a degree that seems unlikely on the species level^12,14^.

X-ray micro computed tomography (µCT) has been a useful method for analysing morphological characters in fossils, especially when specimens are covered by matrix or other material, but flat objects are inherently ill-suited for X-ray CT in general. Here we use propagation phase contrast synchrotron µCT (e.g.^18–20^) on a specimen of *M. bassanii* from the Besano Formation to overcome these problems, as the approach combines the necessary energy and flux to cope with the high-aspect ratio of flat fossils^21^, avoiding complete attenuation in the long axis of the specimen. This is the first time this method has been used to clarify morphology from a fossil from this formation. We were thus able to identify and describe much of the cranial morphology of the specimen and to reconstruct the cranium of this species in a three-dimensional model.

## Material and methods

*Macrocnemus bassanii* specimen PIMUZ T 2477 is one of several specimens in the collections of the PIMUZ. This specimen has previously been described by Peyer^3^. He refers to the specimen as the ‘Cava Tre Fontane’ specimen. The specimen was described and measurements of parts of the cranium and the preserved postcranial elements (which, apart from the axis-atlas complex, will not be discussed here) were provided. Peyer correctly identified the premaxillae and maxillae as well as the frontals, prefrontals and mandible, but had difficulties identifying bones from the posterior skull region and from the palate^3^. This is understandable given the nature of preservation of the specimen in a strongly compressed black shale matrix. PIMUZ T 2477 is better suited than other *Macrocnemus* specimens found in the same locality as its bones are not as crushed as most specimens of any taxon from this locality.

The specimen was scanned at the ID19 beamline of the European Synchrotron Radiation Facility (ESRF, Grenoble, France) using propagation phase contrast synchrotron radiation micro-computed tomography. The experimental setup consisted of: filtered white beam (wiggler W150 gap 26.5 mm, filters: W 2 mm, Cu 6 mm) with a total integrated detected energy of 240 keV, a sample-detector propagation distance of 10 m and an indirect detector (LuAG scintillator 0.33 × magnification from two photographic Hasselblad lenses, sCMOS PCO edge 4.2 Gold) producing data with an isotropic voxel size of 18.21 µm. To image the full sample, the centre or rotation was shifted to increase the lateral field of view by ~ 30%, and 10 acquisition were necessary on the vertical axis (keeping a 50% overlap between consecutive scans). Each acquisition consisted of 6,000 projections of a total integration time of 0.2 s (8 frames of 0.025 s per projection in accumulation mode,^22^) over a rotation of 360°. Before reconstruction, radiographs from the 10 vertical scans were merged together^23^. Tomographic reconstruction was done using PyHST2^24^ using the single distance phase retrieval approach^25^. Post processing included: modification of the bit depth from 32 to 16 bits as a stack of tiff, ring correction^26^; cropping of the volume.

Rendered images were segmented out using the program Mimics Materialise v. 19.0. The produced models of the elements were exported in the Polygon File Format (ply) and imported into the program Blender 2.79 (Blender, Amsterdam, the Netherlands). In Blender, the skull was reconstructed three-dimensionally based on inferred bone contacts and comparisons with closely related taxa such as *Prolacerta broomi*. Images were also rendered in Blender. The 3D model of the cranium, a rendered view of the specimen in situ and a fly-through video of the scan can be found at the ESRF database or as supplementary material added to this publication. The original tiffstack data can be found in the ESRF database (https://paleo.esrf.eu).

Although the skull is largely complete, some bones were missing or only partially preserved. For this reason, some of the bones in the 3D model are mirrored from their symmetric counterparts (see supplementary table S1).

### Institutional abbreviations

BP, Evolutionary Studies Institute (previously Bernard Price Institute for Palaeontological Research), University of Witwatersrand, Johannesburg, South Africa; PIMUZ, Palaeontological Institute und Museum Universität Zürich, Zürich, Switzerland; MSNM, Museo Civico di Storia Naturale Milano, Milano, Italy; GMPKU, Geological Museum of Peking University, Beijing, China; IVPP, Institute for Vertebrate Paleontology and Paleoanthropology, Beijing, China; YPM, Yale Peabody Museum, Yale, USA; ZAR, Zarzaitine collection of the Muséum National d'Histoire Naturelle, Paris, France.

### Systematic palaeontology

Archosauromorpha Von Huene, 1946 ^27^.

Tanystropheidae Camp, 1945 ^6^.

Genus *Macrocnemus* Nopcsa, 1930 ^1^.

*Macrocnemus bassanii* Nopcsa, 1930 ^1^.

**Figure 1:**
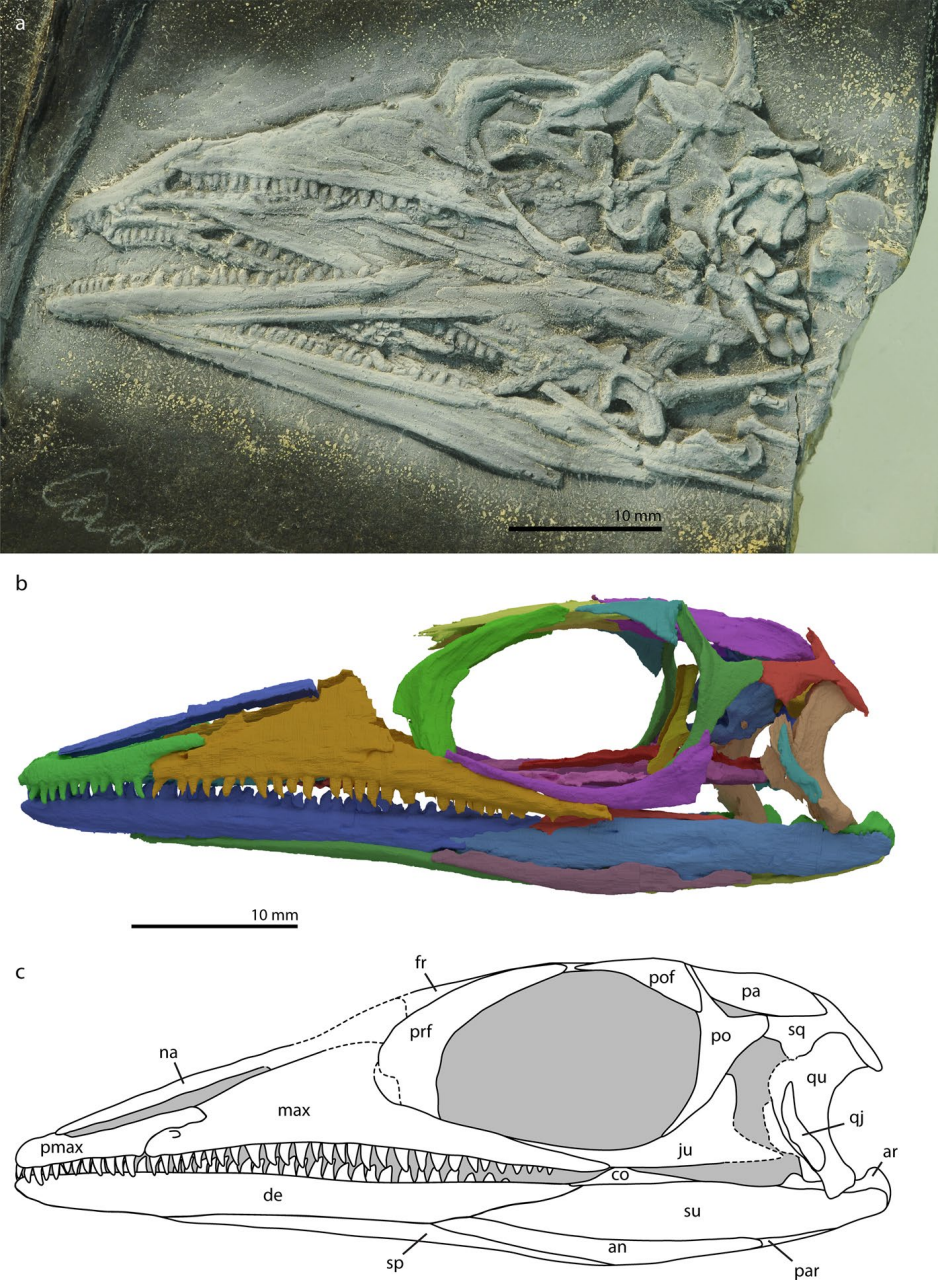
Photograph (**a**), digitally rendered reconstruction (**b**) and life interpretation (**c**) of the cranium of *M. bassanii* specimen PIMUZ T 2477 (**b** and **c** have been mirrored for comparison with **a**). *an* angular, *ar* articular, *co* coronoid, *de* dentary, *fr* frontal, *ju* jugal, *max* maxilla, *na* nasal, *pa* parietal, *par* prearticular, *pmax* premaxilla, *po* postorbital, *pof* postfrontal, *prf* prefrontal, *qj* quadratojugal, *qu* quadrate, *sp* splenial, *sq* squamosal, *su* surangular.

Studied specimen: *M. bassanii*, PIMUZ T 2477 (Fig. 1).

The specimen PIMUZ T 2477 is assigned to *M. bassanii* based on the fore- and hind-limb ratios, which are similar to the other specimens of *M. bassanii* from the Besano Formation including the holotype^12,28^. The extensive cranial morphology here described should aid to further help identifying and developing the cranial characteristics to distinguish between the species of *Macrocnemus*.

### Ontogenetic stage of PIMUZ T 2477

The ontogeny of *Macrocnemus* has received little attention thus far. Authors have referred either to ossification states or to cranial and postcranial ratios to identify differences between juvenile and adult specimens^17,28,29^. The smallest specimen, and so far, the only specimen considered a juvenile, MSNM BES SC 111, is slightly smaller in cranial length than PIMUZ T 2477 (ca. 38 mm and ca. 42 mm respectively). Both specimens are missing the distal tarsal 3^17^ [F.M. pers. observ.], which can be an indication of a juvenile staged individual, as postnatal ossification of tarsal and carpals often occurs in reptiles^17,29,30^. Moreover, the fusion of the transverse processes of caudal and dorsal vertebrae to the centrum has been suggested as characteristic for adult specimens^31^. The transverse processes of PIMUZ T 2477 are fused to the centra (F.M. pers. observ.). It has also been stated that PIMUZ T 2477 falls in range of more adult specimens in terms of cranial ratios, namely the pre-orbital length to cranial length ratio^17^. Juvenile specimens have relatively larger orbits and relatively larger crania compared to adults. Unfortunately, Premru did not disclose how these lengths were measured^17^, which means it is not possible to reproduce the same outcome. On the basis of these observations we consider specimen PIMUZ T 2477 to be closer to the adult stage than to a neonatal stage. Most postnatal cranial changes in the non-archosauriform archosauromorph *Proterosuchus fergusi* are allometric and not qualitative^32^, as is also the case for most extant Squamata^33^. Ontogeny may therefore be partially causing the described morphology of this specimen, but should not represent a very strong factor.

## Results and comparison

### Dermal skull roof

#### Premaxilla

**Figure 2:**
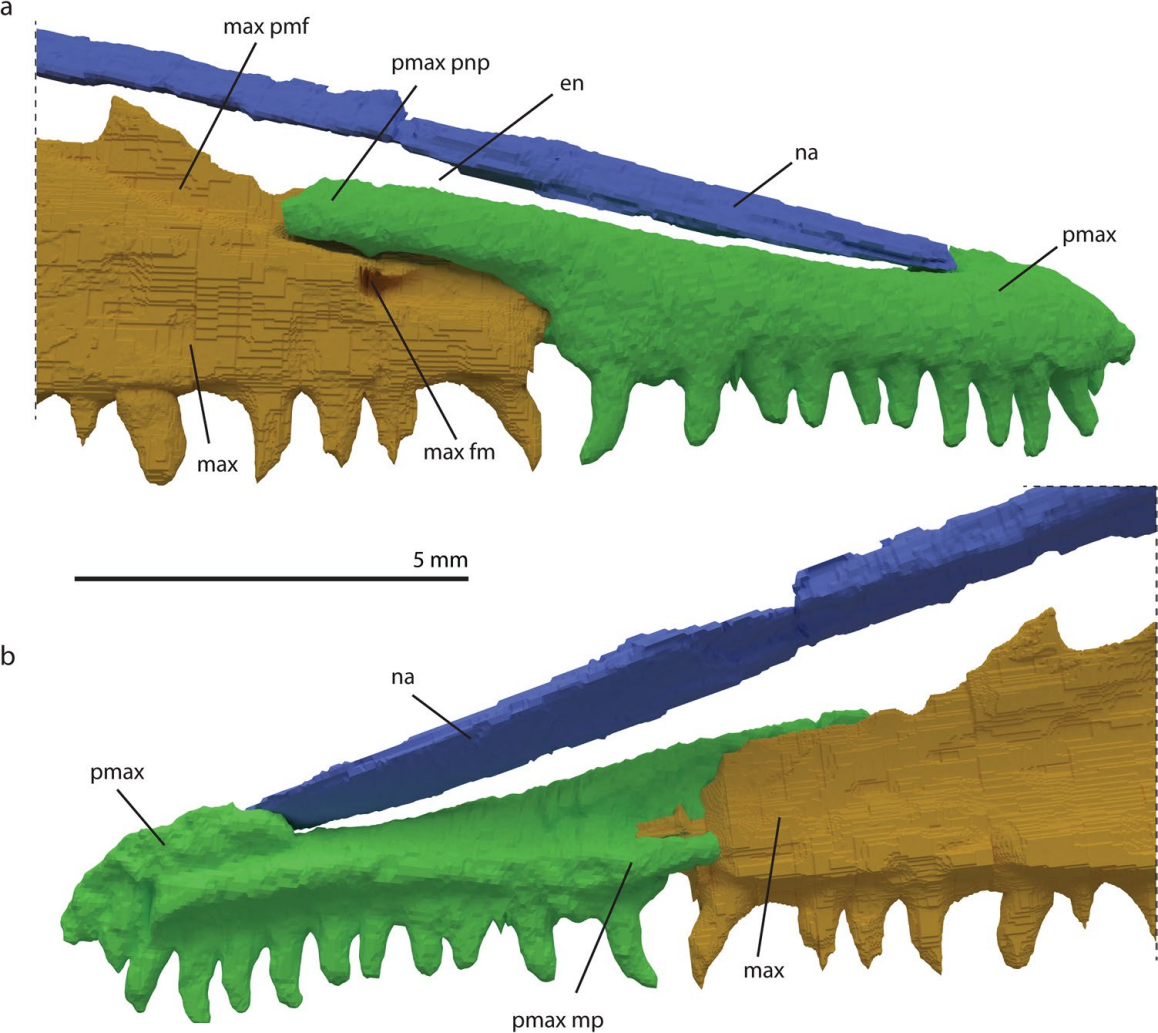
Digitally rendered details of the anterior rostrum of *M. bassanii* specimen PIMUZ T 2477 in lateral view (**a**) and medial view (**b**). *en* external naris, *max* maxilla, *max fm* maxillary foramen, *max pmf* maxillary premaxilla facet, *na* nasal, *pmax* premaxilla, *pmax mp* premaxillary medial process, *pmax pnp* premaxillary postnarial process.

**Figure 3:**
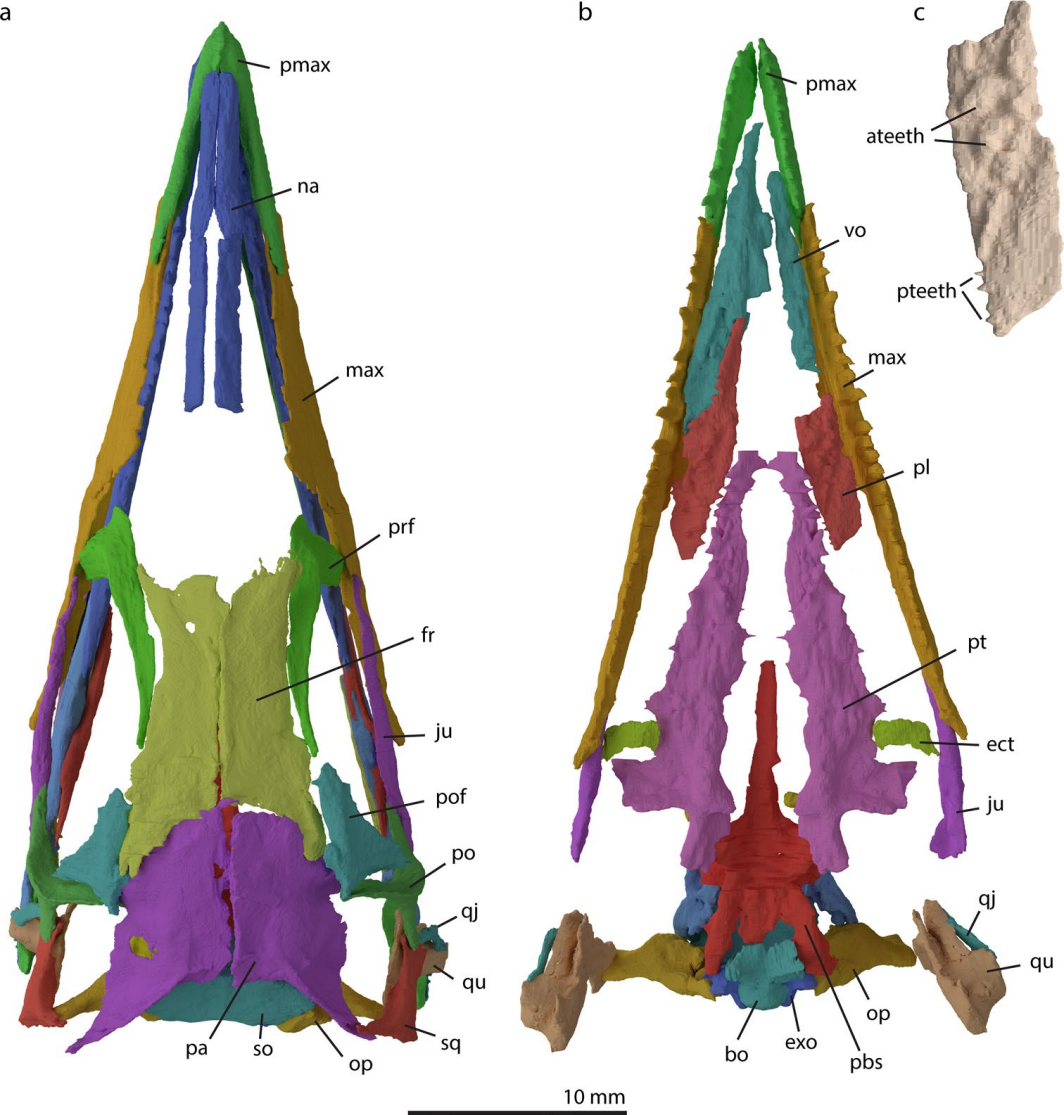
Digitally rendered reconstruction of the cranium of *M. bassanii* specimen PIMUZ T 2477 in dorsal (**a**) and ventral (**b**) view, and detail of the left palatine in ventral view (**c**, not to scale). Note that the bones of the palate have been removed in **a** and the focus lies on the palatal bones in **b**. The palatine shows antero-posterior heterodonty, whereby anteriorly the teeth are more bulbous and ventrally directed, whereas posteriorly they are slenderer and medially directed. *ateeth* anterior teeth, *bo* basioccipital, *ect* ectopterygoid, *exo* exoccipital, *fr* frontal, *ju* jugal, *max* maxilla, *na* nasal, *op* opisthotic, *pa* parietal, *pbs* parabasisphenoid, *pl* palatine, *pmax* premaxilla, *po* postorbital, *pof* postfrontal, *prf* prefrontal, *pt* pterygoid, *pteeth* posterior teeth, *qj* quadratojugal, *qu* quadrate, *so* supraoccipital, *sq* squamosal, *vo* vomer.

The premaxilla is the anterior-most element in the rostrum. It is a slender, antero-posteriorly elongated element. It articulates with the maxilla posteriorly, with the nasal dorso-medially, whereby it forms the lateral side of the external naris (Fig. 1), and with the vomer ventro-medially. Both premaxillae are fully preserved and almost in articulation with their respective maxillae in PIMUZ T 2477. The premaxillae touch at the midline, but do not form an extended symphyseal structure. Antero-dorsally the prenarial process of the premaxilla is present as a small convex process, on which it contacts the anterior-most margin of the nasal. The prenarial process is generally pronounced in early archosauromorphs, but is also poorly developed or absent in rhynchosaurs, *Azendohsaurus madagaskarensis*, and *Tanystropheus longobardicus*^7,11,34^ (Figs. 2, 3). The premaxilla has two additional, more prominently visible, elongate processes which are directed posteriorly. The dorsal one of these two, the postnarial process, articulates with a groove on the antero-dorsal side of the maxilla (Fig. 2a). The ventral or maxillary process extends as a ridge along the medial side of the premaxilla to the maxilla, just dorsally of the tooth alveoli (Fig. 2b). It extends further posteriorly than the last tooth position and articulates with the medial side of the maxilla. Although a small additional projection on the posterior part of the premaxilla is known in *Azendohsaurus madagaskarensis* and *Erythrosuchus africanus*^34,35^, the ventral process of *M. bassanii* differs distinctly from the process in these taxa in it being positioned on the medial side of the premaxilla and therefore not visible in lateral view when in articulation with the maxilla. In that regard, the ventral process of *M. bassanii* is more similar to the palatal process as previously identified in archosauriforms (e.g. *Garjainia prima*^36^). However, we infer that in *M. bassanii* this process likely did not contribute to the palate but instead articulated tightly on the medial surface of the anterior part of the maxilla (Fig. 2b).The premaxilla has 12–13 tooth positions; the teeth are homodont in shape, not labio-lingually compressed and slightly recurved (Fig. 1).

The postnarial process of the premaxilla of *M. bassanii* has been described earlier (e.g.^8,17^). This process is common among early diverging archosauromorphs and is prominent in for instance *Azendohsaurus madagaskarensis, Prolacerta broomi* and early Archosauriformes, for example: *Garjainia prima* and *Proterosuchus fergusi*^32,34,36,37^. In all those taxa the postnarial process extends more dorsally and contacts the nasal, restricting the antero-posterior extension of the external naris^32,34,36,37^. This is not the case in *M. bassanii*, making the external naris elongated and slit-like. *Protorosaurus speneri* and the large morphotype of *Tanystropheus longobardicus* lack a postnarial process entirely, however it is present to a degree in the small morphotype^7,16,38^.

#### Maxilla

The maxilla forms the largest part of the lateral, pre-orbital region of the cranium (Fig. 1). It is a triangular structure with a slightly concave posterior margin. Both maxillae are present in this specimen. The left maxilla is broken postero-dorsally and the right maxilla has been crushed, which made it difficult to discern its original morphology. The maxilla articulates with the premaxilla anteriorly, the prefrontal, and likely the lacrimal, posteriorly, the jugal postero-ventrally, and likely the palatine postero-medially (Figs. 1, 3). A clear facet is visible along the anterior portion of its dorsal margin in the form of a groove that anteriorly articulated with the postnarial process of the premaxilla. Directly ventral to this facet, an anteriorly projecting maxillary foramen is present with a short groove extending anteriorly (Figs. 1,  2a). Such a maxillary foramen is present across early diverging Archosauromorpha for example in *Protorosaurus speneri*, *Prolacerta broomi* and *Azendohsaurus madagaskarensis* and a groove is present in the same position in *Garjainia prima*^34,36–38^. A foramen is not present in *Tanystropheus longobardicus* and *Mesosuchus browni*^7,11^. The premaxilla also articulates with the maxilla antero-medially, but a facet for this articulation on the maxilla is not apparent (Fig. 2b). The ascending process of the maxilla inclines postero-dorsally in a roughly 35°–40° angle. Based on the maxillae of other specimens of *M. bassanii* (e.g. PIMUZ T 4822; see Fig. 10 in^12^), the dorsal margin of the maxilla would have ended abruptly posteriorly resulting in a concave posterior margin of the maxilla, similar to other early archosauromorphs. This morphology is present to a lesser degree in *Prolacerta broomi*, *Protorosaurus speneri* and *Mesosuchus browni*, whereas it is extremely apparent in *Azendohsaurus madagaskarensis*^11,34,37,38^.

Posteriorly the maxilla articulates with the prefrontal, whereby the latter either touches the posterior edge, or overlaps the postero-lateral margin of the maxilla slightly (Fig. 1). Postero-ventrally the posterior process of the maxilla tapers to a point. It has a large groove that forms a facet for the jugal on its dorso-medial surface. This groove aligns with almost the entire length of the anterior process of the jugal, giving the impression that the maxilla is a large contributor to the orbit ventrally in lateral view (as seen in for instance PIMUZ T 4822;^12^). The best-preserved maxilla has 23 teeth present in situ, but there is space to accommodate approximately 30 teeth, extending as far posteriorly as half the antero-posterior length of the orbit. The anterior teeth are more or less similar to those in the premaxilla, but in the mid-section they are slightly less recurved and more robust than the premaxillary teeth. There is no labiolingual compression (Fig. 1).

#### Nasal

**Figure 4:**
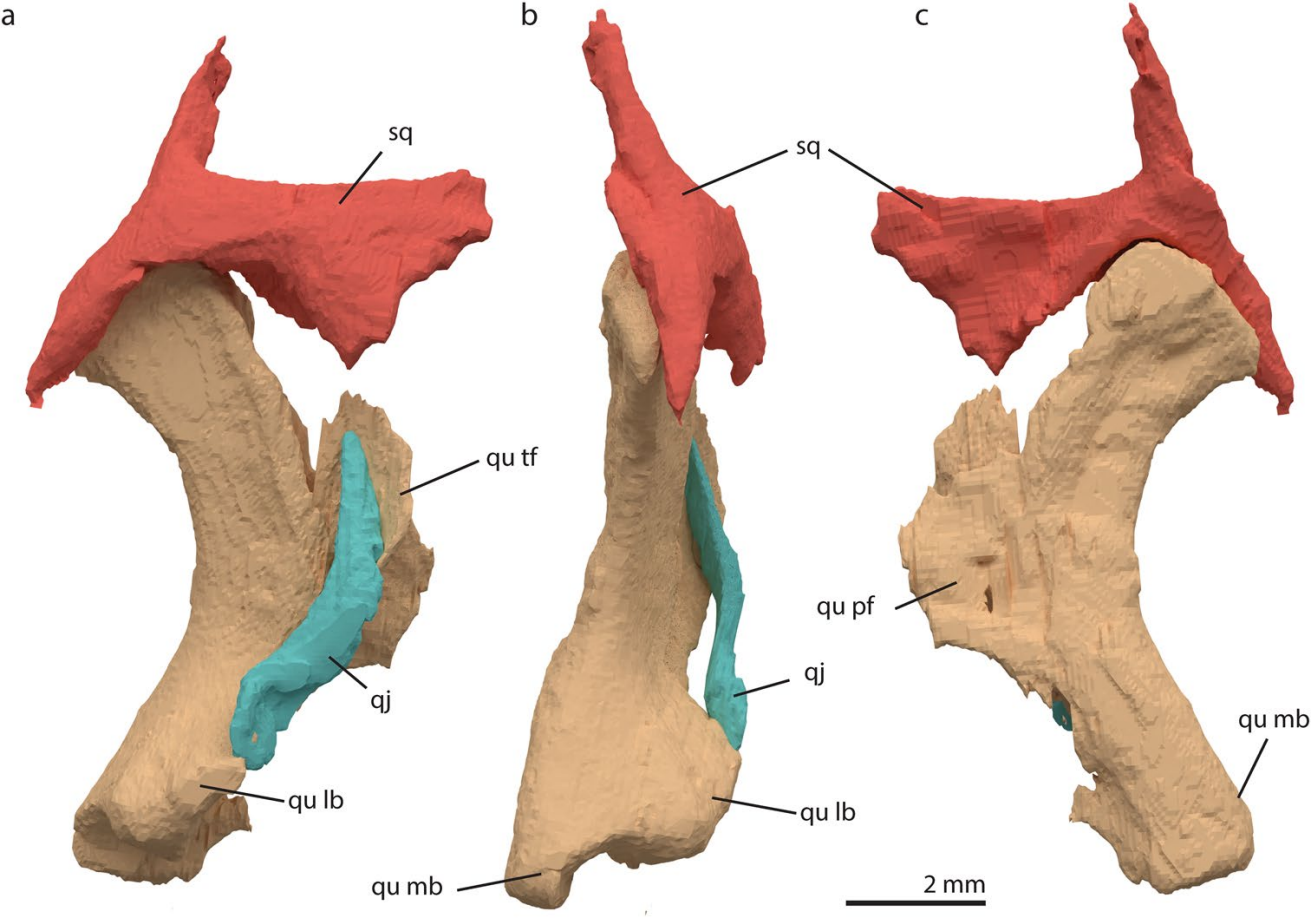
Digitally rendered details of the left quadrate-squamosal joint of *M. bassanii* specimen PIMUZ T 2477 in lateral view (**a**), posterior view (**b**), and medial view (**c**). Note tight fit of the quadrate head and the quadrate articulation facet on the squamosal in **A**. *qj* quadratojugal, *qu lb* quadrate lateral boss, *qu mb* quadrate medial boss, *qu pf* quadrate pterygoid flange, *qu tf* quadrate tympanic flange, *sq* squamosal.

The nasals have unfortunately been poorly preserved in this and all other specimens with the exception of PIMUZ T 1559 (Fig. 4 in^12^). We did identify medially situated and elongated rod-like parts of bone in PIMUZ T 2477 (Figs. 2a,b, 3) that are herein both referred to as the premaxillary processes of the nasal sensu Jaquier et al.^12^. This lends support to their identification of the nasal being a combination of a posteriorly flat surface and a more rod-like anterior premaxillary process, as that morphology would accommodate the frontals posteriorly and form the internarial bar anteriorly. The anterior slenderness of the premaxillary process of the nasal and the orientation of the postnarial process of the premaxilla result in a very elongated slender external naris thus more resembling extant varanids rather than other non-archosauriform archosauromorphs^3^.

#### Jugal

The jugal forms most of the ventral side of the orbital margin. Its anterior process is an elongate antero-dorsally curving structure that almost reaches the anterior extent of the orbit (Fig. 1b). Both jugals are present in the specimen described here. The left jugal is nearly complete and little altered; the right jugal is completely broken and crushed by other bones. The left jugal is disarticulated, but is in close proximity to its life position. The anterior process of the jugal fits in the grooved facet of the maxilla and the ascending process of the jugal articulates postero-dorsally with a groove on the posterior surface of the ventral process of the postorbital (Fig. 1). The posterior process of the jugal, which would complete its tripartite morphology, is not preserved in PIMUZ T 2477, but has been observed in other specimens (e.g. PIMUZ T 1559 and PIMUZ T 4822; Figs. 4 and 10 in^12^). From these specimens it can be inferred that the process is short, and abruptly tapering, which leads to our reconstruction of an open ventral emargination, as was discussed in detail by Rieppel and Gronowski^39^. An incomplete lower temporal bar is a typical non-archosauriform archosauromorph trait, and the jugals of most early archosauromorphs have a short posterior process, with the exception of *Trilophosaurus buettneri*, which lacks an infratemporal fenestra, and *Dinocephalosaurus orientalis* and *Pectodens zhenyuensis*, which both lack a posterior process of the jugal^40–42^.

#### Prefrontal

The prefrontal forms the anterior and antero-dorsal portions of the orbital margin. It is a crescent-shaped element, articulating with the maxilla and almost certainly the lacrimal anteriorly, the frontal dorso-medially, and likely the nasal antero-dorsally (Fig. 1). The prefrontal is very elongate posterodorsally, forming much of the anterior and dorsal margin of the orbit. In our reconstruction, the prefrontal almost touches the anterior portion of the postfrontal. Both elements thereby leave a small margin for the frontal to contribute to the orbit. Dorsally the prefrontal is flat; this area is inferred to form the frontal facet. The element thickens antero-laterally forming the anterior side of the orbit (Fig. 1). Both prefrontals are well preserved in PIMUZ T 2477.

#### Lacrimal

Due to the compacted nature of the skull, a lacrimal in articulation with the prefrontal was not encountered in PIMUZ T 2477. However, fragmentary elements preserved in close vicinity to the prefrontal and posterodorsal margin of the maxilla could pertain to the lacrimal. In our reconstruction, we noticed a space anterior to the prefrontal and postero-dorsal to the maxilla. As stated, the posterior margin of both maxillae in PIMUZ T 2477 is broken or crushed and a small lacrimal is present in this space (Fig. 1b; but, see also Fig. 10 in^12^). A small lacrimal is generally present ventral to the prefrontal and posterior to the ascending process of the maxilla in non-archosauriform archosauromorphs (e.g. *Prolacerta broomi*, *Tanystropheus longobardicus*, *Dinocephalosaurus orientalis*, *Pectodens zhenyuensis*, and several allokotosaurs and rhynchosaurs^11,34,37,41–43^).

#### Postfrontal

The postfrontal is a triangular element that forms the postero-dorsal margin of the orbit (Figs. 1, 3). We observed only the left postfrontal in PIMUZ T 2477. In our reconstruction, it contacts the parietal along most of its dorsal margin, with only the anterior-most portion of the dorsal margin contacting the frontals (Fig. 3). On its posterior margin, the postfrontal articulates with the dorsal process of the postorbital. The antero-ventral margin accommodates the orbit. Ventro-medially, the element is slightly concave. In this configuration, the postfrontal and its articulation with surrounding bones is virtually identical to *Prolacerta broomi*^37,44^. Due to the incomplete preservation of the anterior process, it cannot be established with certainty whether the bone contacted the posterior aspect of the prefrontal or if both elements were separated, although specimen PIMUZ T 4822 appears to indicate that they did not touch (see also^12^). We therefore reconstruct the frontals to form part of the dorsal region of the orbital margin.

#### Postorbital

The postorbital is a triradiate bone that forms the posterior margin of the orbit. Antero-dorsally the element articulates with the postfrontal (and possibly the parietal), antero-ventrally with the jugal and posteriorly with the squamosal (Fig. 1). The element is antero-medially concave and thickened to accommodate for the posterior rim of the orbit. The posterior process articulates with a facet on the anterior process of the squamosal and the ventral process bears a clear groove that received the ascending process of the jugal. The posterior process of the postorbital forms the anterior part of the lateral margin of the upper temporal fenestra, as well as the majority of the anterior and part of the dorsal margin of the lower temporal fenestra. This is also very similar to the morphology seen in *Prolacerta broomi*^37,44^, whereas it differs from other tanystropheids and closely related taxa, in which the postorbital more extensively formed the ventral margin of the upper temporal fenestra (*Tanystropheus longobardicus*, *Dinocephalosaurus orientalis*, *Pectodens zhenyuensis*^41–43^).

#### Squamosal

The squamosal is a complex element with four processes, articulating with the postorbital anteriorly, the opisthotic and parietal dorso-medially and with the quadrate ventrally (Figs. 1, 3, 4). Only the left squamosal is preserved in this specimen; it is disarticulated, but in close association with the quadrate and the postorbital. Antero-laterally, the squamosal has an articular surface on which the posterior process of the postorbital articulates (Figs. 1, 4A). Postero-ventrally the squamosal has a large concave articulation surface on its medial side where the quadrate articulates (Fig. 4B). Directly anterior to the quadrate facet, at the base of its anterior process, the squamosal bears a short, possibly incomplete ventral process that would have covered the quadrate anteriorly, with little lateral or medial overlap. The posterior process of the squamosal forms part of the articulation surface for the dorsal head of the quadrate and protrudes distinctly posterior to it, similar to *Protorosaurus speneri*, *Prolacerta broomi*, and *Eohyosaurus wolvaardti* among non-archosauriform archosauromorphs^37,38,45^. The dorso-medial process, which articulates with the parietal, has a concave facet postero-medially on the proximal part. This could be the facet for the articulation with the paroccipital process of the opisthotic. The squamosal also forms the posterior and lateral margins of the upper temporal fenestra and the postero-dorsal margin of the lower temporal fenestra (Fig. 1). Due to the rather fragile nature of the squamosal, the element (especially the thin dorsal process) has experienced some deformation, which renders a tight articulation with the parietals difficult in the 3D model.

#### Quadratojugal

The quadratojugal is an s-shaped elongated bone in posterior view and is slightly semilunar-shaped in lateral view. Both elements are preserved. The left element was observed in association with the quadrate, the right element was disassociated from the corresponding quadrate. Its ventral portion likely articulates on the dorsal surface of the latero-ventral boss of the quadrate and the dorsal side on the lateral side of the tympanic flange of the quadrate (Fig. 4). This is the first unequivocal identification of a quadratojugal in tanystropheids, although its presence is widespread among other non-archosauriform archosauromorphs (e.g. *Prolacerta broomi*, *Azendohsaurus madagaskarensis*, *Mesosuchus browni*^11,34,37^). We deem it unlikely that the quadratojugal contacts the squamosal dorsally given its size, even if the ventral margin of the squamosal is incomplete, which could be the case.

#### Quadrate

Only the left quadrate is preserved in PIMUZ T 2477. The element is strongly and continuously curved in lateral view, with a smooth concave posterior margin and a convex anterior margin (Figs. 1, 4), roughly resembling the morphology of *Prolacerta broomi*^37^. The dorsal head articulating with the squamosal has a slight posterior expansion but lacks a hook as seen in allokotosaurs (e.g. *Azendohsaurus madagaskarensis*^34^). The dorsal head lacks any clear medio-lateral expansion and fits in the concave articulation surface on the ventro-medial side of the posterior part of the squamosal (Fig. 4B). From the medial surface at about mid-height of the bone, the pterygoid flange protrudes from the quadrate. The flange is relatively short and does not reach the dorsal margin of the main body of the quadrate as seen in *Azendohsaurus madagaskarensis*^34^. The orientation of the pterygoid flange is exclusively anterior; however, it is unclear whether this is due to compression of the element and it would have had a more antero-medial orientation in life as in other archosauromorphs^46^. A larger ellipsoidal flange protrudes antero-laterally; this is the tympanic flange. Together with the curved morphology of the quadrate the size of the flange strongly suggests that this is the position of the tympanic membrane as occurs in most other diapsids^47^. In PIMUZ T 2477, the tympanic flange was slightly broken off from its original position, but remains almost articulated to the main body of the quadrate. The two flanges extend relatively parallel to the main body of the quadrate, thereby forming a groove along the anterior side of the quadrate. The quadrate ends in two lateral bosses ventrally, which form the articular condyle. The medial-most of these two bosses reaches further ventrally than the lateral one. In our reconstruction the orientation of the quadrate is almost strictly dorso-ventral, whereby the ventral condyles do not lie more posterior than the dorsal head. This orientation is likewise seen in *Protorosaurus speneri* and *Azendohsaurus madagaskarensis*^34,38^. The ventral condyles lie slightly more posterior in *Prolacerta broomi* and distinctly more posterior in Erythrosuchidae^32,36,37^.

#### Frontal

The paired frontals show a midline suture and are thus unfused. In general, the frontal is an elongated flattened element that forms the dorsal rim of the orbit and the anterior section of the roof of the cranium (Figs. 1, 3). Both frontals are relatively broad anteriorly, and then become narrower over the interorbital region, before broadening again posteriorly ending in two distinct posterolateral processes, forming a u-shaped contact with the parietal. This contact is considered as an autapomorphy of the genus *Macrocnemus*^9,12^. Anteriorly both frontals are slightly broken, but it seems that they display small facets for the articulation with the nasals on their dorsal surface antero-medially. Antero-laterally, the frontal articulates with the prefrontal on its ventral surface. Posteriorly, the frontal has an extending robust process postero-laterally that encloses the antero-lateral side of the parietal, thereby articulating with it. In ventral view, a large triangular indentation, which is formed by both frontals and bordered laterally by the edges of the ventral side of the parietal flanges, can be observed (Fig. 6). More anteriorly, the frontals each have a smaller indentation separated by a ridge that extends along the midline. These indentations accommodate the olfactory lobes. This morphology is shared with *Tanystropheus longobardicus* and is possibly a synapomorphy of the tanystropheid family (see character 121 of Ezcurra^9^). The larger posterior triangular indentation would have covered the forebrain^48^. This interpretation is supported by the circumstance that there is a high conservation of the relative position of the frontal and parietal and which brain sections they cover across Diapsida^48^.

#### Parietal

**Figure 5:**
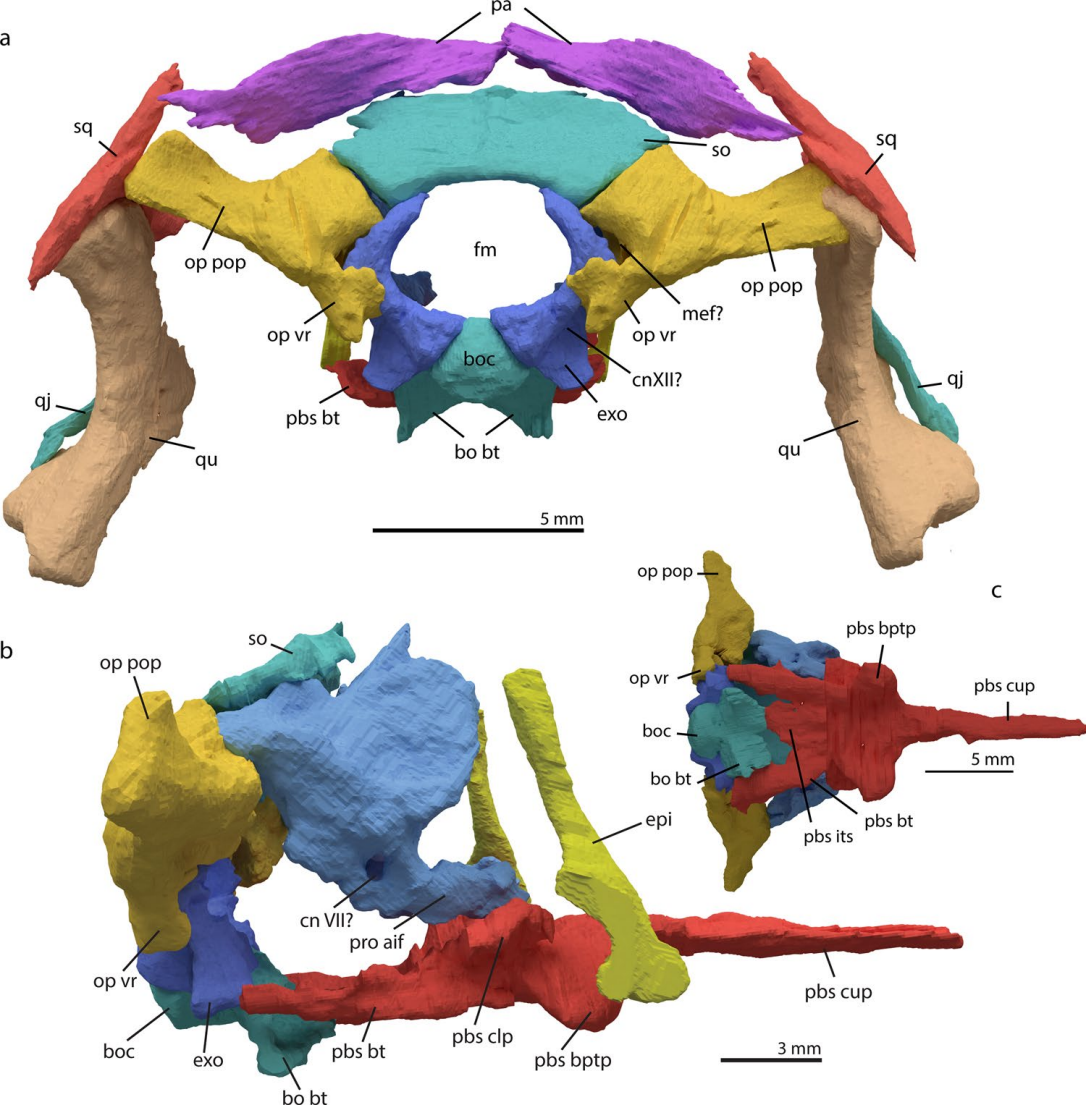
Digital rendering of the reconstructed cranium of *M. bassanii* specimen PIMUZ T 2477 in occipital view (**a**), braincase in right lateral view (**b**) and braincase in ventral view (**c**). *bo bt* basioccipital basal tubera, *boc* basioccipital condyle, *CN VII?* possible position of the foramen of cranial nerve VII, *CNXII?* possible position of the hypoglossal foramen, *epi* epipterygoid, *exo* exoccipital, *fm* foramen magnum, *mef?* hypothetical position of the metotic fissure, *op pop* opisthotic paroccipital process, *op vr* opisthotic ventral ramus, *pa* parietal, *pbs bptp* parabasisphenoid basipterygoid process, *pbs bt* parabasisphenoid basal tuber, *pbs clp* parabasisphenoid clinoid process, *pbs cup* parabasisphenoid cultriform process, *pbs its* parabasisphenoid intertuberal surface, *pro aif* prootic anterior inferior process, *qj* quadratojugal, *qu* quadrate, *so* supraoccipital, *sq* squamosal.

Both parietals are present in PIMUZ T 2477. The parietals are unfused, as in the tanystropheid *Tanytrachelos ahynis* (YPM 7,482), *Jesairosaurus lehmani* (ZAR 07), *Prolacerta broomi*, and allokotosaurs among non-archosauriform archosauromorphs (Fig. 3)^34,37,40,49,50^. They are well preserved and in close association with each other. The parietals are located directly posterior to the frontals in the skull roof. The parietal is a flat quadrangular element with a process projecting postero-laterally. Anteriorly, there appears to be a facet for the reception of the frontal on the dorsal surface of the parietal, indicating that the frontal partially overlapped the parietal dorsally. Laterally, the parietal contacts the postfrontal and likely the dorsal-most part of the postorbital. Posteriorly, the parietal articulates with the supraoccipital and its postero-lateral flange articulates with the squamosal and possibly the paroccipital process of the opisthotic. Due to the morphology of the supraoccipital and posterior margin of both parietals, we hypothesize a tight articulation and therefore exclude the possibility of postparietals being present (Fig. 5), as is the case for all non-archosauriform archosauromorphs in which this could be assessed. Similarly, the absence of discernible supratemporal bones in this specimen or any other known *Macrocnemus* specimens, and the apparently neat fit between the squamosal and parietal, indicate that the supratemporals were absent as separate ossifications. Supratemporals are known to occur in most non-archosauriform archosauromorphs except for allokotosaurs^34,51^. The supratemporal was previously tentatively identified in *Tanystropheus longobardicus*, but this identification is equivocal^7,43^. The supratemporal fossae, which are present on the proximo-anterior margin of each posterior flange, are weakly developed. There is no evidence of a parietal foramen (Fig. 3), nor is there indication of the presence of such a foramen in any other *Macrocnemus* specimen described to date. The presence of the parietal foramen is intraspecifically variable in *Prolacerta broomi*^37^, and *Proterosuchus fergusi*, whereby in the latter there is evidence for ontogenetic variation specifically^32^. In ventral view, round indentations are visible on both parietals symmetrically (see below). In extant squamates, these are considered to accommodate posterior brain parts such as the optic lobe, cerebellum and parts of the cerebrum^52^. Moreover, in Archosauria the parietal covers the midbrain, which includes the optic lobes and posterior portions of the cerebrum^48^. We can therefore infer that this is also the case in *Macrocnemus*.

### Braincase

#### Basioccipital

The basioccipital articulates with the exoccipitals dorso-laterally and with the parabasisphenoid anteriorly (Fig. 5). Dorso-laterally, it has two facets on either side to accommodate the exoccipitals. Anteriorly, the basioccipital has two triangular facets and two triangular indentations ventral to them. We interpret these structures as the combined basisphenoid facet. This facet is very similar to what is described for *Prolacerta broomi*^53^, but cannot be observed in taxa with a fused basioccipital-parabasisphenoid, as in most Archosauriformes^54^. The parabasisphenoid and basioccipital do not fit together precisely and the basal tubera are not connected by an intertuberal plate in the parabasisphenoid as in other non-archosauriform archosauromorphs. However, it is unclear whether this portion of the element could not be discerned on the synchrotron µCT data, or whether the posterior part of the parabasisphenoid was incompletely ossified. The condylar part of the basioccipital is circular ventrally and is slightly triangular in the more dorsal region. The triangular morphology results from the dorso-lateral sides of the condyle also serving as the posterior facets for the exoccipitals and the exoccipitals contributing to the occipital condyle (Fig. 5a). The dorsal-most (sagittal) surface of the condyle forms the floor of the foramen magnum. No notochord pit is present on the condyle as in other early archosauromorphs including *Prolacerta broomi*, *Mesosuchus browni* and *Euparkeria capensis*^46,53,55^. The distance between the basal tubera of the basioccipital is roughly similar in width to the condyle. The tubera are located distinctly ventro-lateral to the condyle and meet medially under the condyle forming a concave structure (Fig. 5). Their contact through a horizontal ridge is more extensive than in *Prolacerta broomi*, in which this ridge is virtually absent^53^. More similar than *Prolacerta broomi* to *M. bassanii* is *Mesosuchus browni* as it displays a similar concave structure^46^.

#### Parabasisphenoid

**Figure 6:**
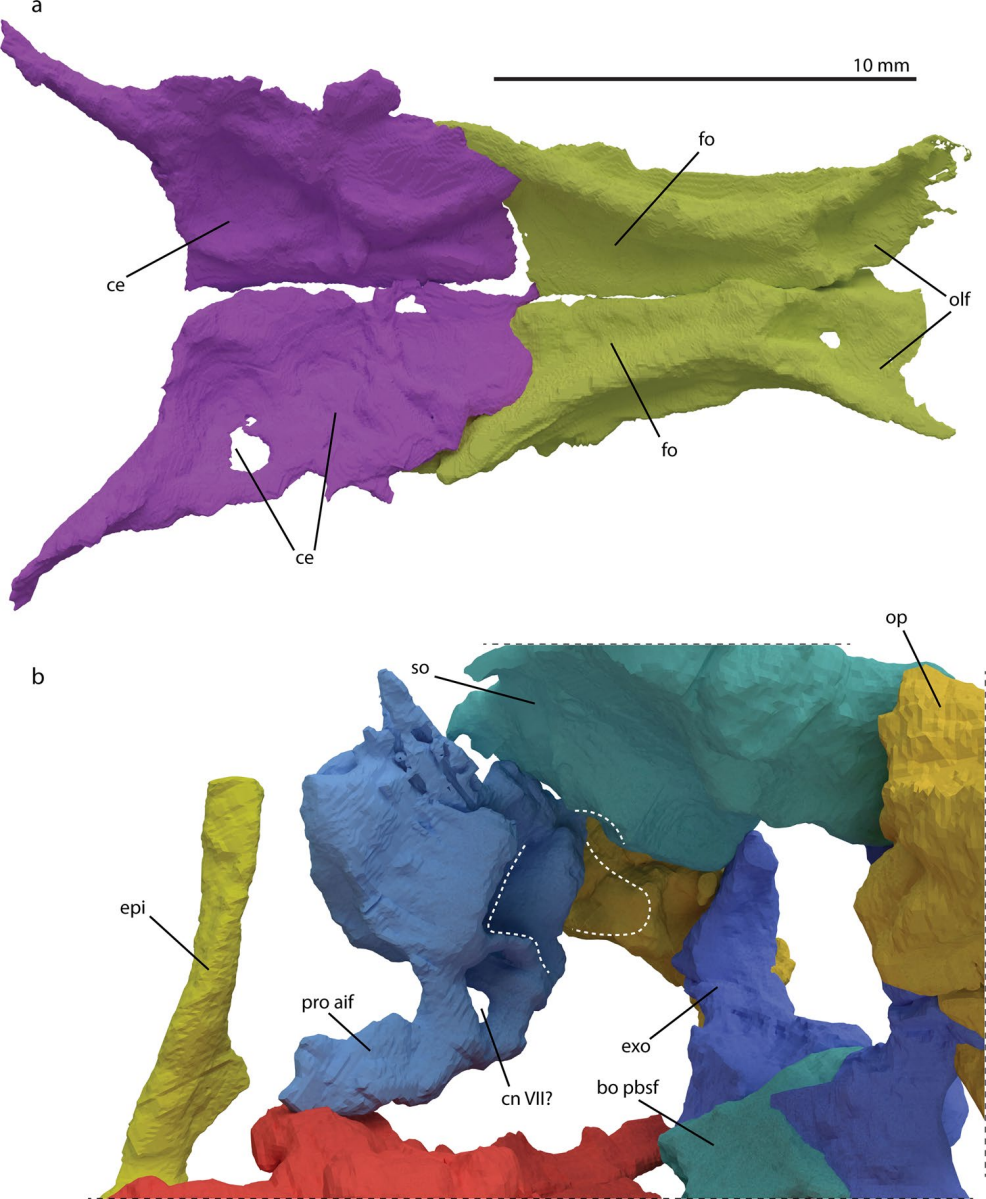
Digitally rendered frontals and parietals of *M. bassanii* specimen PIMUZ T 2477 in ventral view reconstructed in hypothetical life position (**a**) and braincase in antero-medial view (**b**). *bo pbsf* basioccipital parabasisphenoid facet, *ce* indentations of the (posterior) cerebral hemispheres, *cnVII?* hypothetical position of cranial nerve VII, *epi* epipterygoid, *exo* exoccipital, *fo* indentations of the anterior cerebral hemisphere (forebrain), *olf* indentation of the olfactory lobes, *op* opisthotic, *pro aif* prootic anterior inferior process, *so* supraoccipital.

The parabasisphenoid consists of the fused parasphenoid and basisphenoid and connects with the basioccipital posteriorly, the prootics dorso-laterally, and the pterygoids ventro-laterally (Figs. 3, 5b). The posterior portion shows a thin, flat surface medially alongside two postero-laterally projecting basal tubera. This surface is incomplete in PIMUZ T 2477. The articulation surface with the basioccipital is not preserved, but the anterior margin of the basioccipital seems to indicate that the bones were connected through a simple, unfused suture (Fig. 6 and supplementary material). Anterior to the basal tubera, the main body of the parabasisphenoid displays four different processes. The posterior of these are the clinoid processes; they project dorso-laterally and contact the prootics (Figs. 5b, 6b). Posterior to the clinoid processes, there appears to be no other connection to the prootics, indicating that the ventral part of the braincase was not fully ossified as in *Youngina capensis* (Fig. 5b)^56^. The clinoid processes are round, but flatten dorsally to accommodate the anterior inferior process of the prootics. Anterior to the clinoid processes and on the ventrolateral surface of the parabasisphenoid are the ventro-laterally projected basipterygoid processes. The basipterygoid processes are rounder and larger than the clinoid processes and do not display an as extensive facet for articulation as the clinoid processes. The basipterygoid processes do not extend as far ventrally as in *Mesosuchus browni*, but they are more prominent than in *Prolacerta broomi*^46,53^. The size and roundness indicate an articulation over a large surface between the parabasisphenoid and pterygoid. It may also indicate motility in the posterior palatal region to some degree^39^. Just anterior to the basipterygoid processes on the dorsal surface of the parabasisphenoid lies the hypophysial fossa, which is a triangular ridge bordering an indention in the parabasisphenoid (supplementary material). This trench is continuing in the anterior cultriform process (Fig. 5), which is elongate and straight, and tapers to a point, with a dorsal trench, making it V-shaped in cross section, similar morphology has been observed in *Prolacerta broomi* and *Mesosuchus browni*^46,53^. As in all other known archosauromorphs, the cultriform process does not bear any dentition. There is no dorso-ventral constriction at the base of the cultriform process as seen in *Euparkeria capensis*^55^. The process extends medially to the palatal rami of the pterygoids. Due to taphonomic flattening of the bone, the foramina of the internal carotid arteries or potential foramina for cranial nerve VI could not be discerned in the synchrotron µCT data. No distinct foramina were likewise described for PIMUZ T 1559, which might suggest that these structures are absent or very small^12^. In other Permo-Triassic diapsids such as *Prolacerta broomi* and *Youngina capensis* the foramina are paired and dorsally located between the basipterygoid processes anteriorly to the dorsum sellae^53,56^. Ventrally the foramina are visible in *Prolacerta broomi* between the basipterygoid processes^37,44^. Likewise, the morphology of the intertuberal plate is similar to that in *Prolacerta broomi*^53^. It is a thin sheet-like structure with an irregular posterior margin. The plate may be distorted somewhat in PIMUZ T 2477, but the same morphology is visible in PIMUZ T 1559^12^.

#### Exoccipital

The exoccipitals are not fused to any other braincase elements (Fig. 5). Ventrally they have two foot-like expansions, which contact the anterior and posterior facets on either side of the basioccipital, whereby the posterior feet contribute to the occipital condyle, making it tripartite (Fig. 5a). The posterior feet do not meet at the midline, thereby including the basioccipital to form the margins of the foramen magnum. This morphology is very common in archosauromorphs, but notably absent in *Erythrosuchus africanus*^57^. The contribution of the exoccipitals to the basioccipital condyle is relatively common among saurians, similar morphology is observed in *Prolacerta broomi*, *Euparkeria capensis*, and many extant squamates^33,53,55^, but not in the non-saurian diapsid *Youngina capensis*^56^. Only in the small morphotype of *Tanystropheus longobardicus* can this character be observed, since it can be inferred from the morphology of the basioccipital that the exoccipital also contributed considerably to the occipital condyle^7^. The exoccipitals of *M. bassanii* are roughly crescent-shaped, their arches facing each other dorso-medially, but they do not touch at the midline here either. In this way, the exoccipitals contribute to the lateral and most of the ventral side of the foramen magnum, but to a lesser degree to the dorsal margin (Fig. 5a). Dorsally the exoccipitals are in contact with the supraoccipital and antero-laterally with the opisthotics (Fig. 6b). There are no apparent foramina in either exoccipital for the passage of the hypoglossal CN-XII. However, this may be a preservation artefact as there are grooves apparent on the expected position for these foramina on the external surface of the elements (Fig. 5 and supplementary material). The foramen was almost certainly present as a separate opening since this occurs across Diapsida (e.g.^53,57,58^). Furthermore, the position of a groove corresponds to the location of the foramen similar to that described for *Prolacerta broomi*^53^.

Fusion of the exoccipitals to the basioccipital and/or the opisthotic is common among Archosauromorpha. In most Erythrosuchidae and crown-group archosaurs the exoccipital is fused with both the basioccipital and opisthotic (and subsequently to the rest of the braincase), whereby no suture lines are observable, as for example seen in *Euparkeria capensis*, *Garjainia prima* and Crocodylia^36,55,59^. In *Mesosuchus browni,* the exoccipitals are fused to the basioccipitals and in tight connection to the opisthotics^46^, whereby a clear suture line is visible between the exoccipitals and the opisthotics. In *Prolacerta broomi,* the exoccipitals and opisthotics are unfused, but instead the exoccipitals are fused to the basioccipital^53^. The overall shape of the exoccipitals of *M. bassanii* is made up of an extended dorsal sheet and slender dorso-ventral mid-section, a broad ventral section contributing ventromedially to the occipital condyle, and a medially arched dorsal portion. This morphology resembles *Prolacerta broomi* and *Mesosuchus browni*^46,53^. A fully unfused braincase is uncommon among non-archosauriform archosauromorphs and is usually a feature of non-saurian diapsids such as *Youngina capensis*^56^. The morphology of *M. bassanii* is notably different from *Tanystropheus longobardicus*, in which the exoccipitals are clearly fused to the opisthotics, but not to the basioccipital^7^.

#### Supraoccipital

The supraoccipital is the dorsal-most structure in the posterior braincase. It contributes to the dorsal side of the foramen magnum by contacting the exoccipitals medio-ventrally (Figs. 5a, 6b). The supraoccipital thickens ventro-laterally. This thickening likely forms the contact point for the opisthotics on either side (Figs. 5a, 6b and supplementary material). In antero-ventral view, a distinct concavity is visible on the thickened lateral ends of the supraoccipital, which represents the contribution of the supraoccipital to the inner ear capsule (Fig. 6b). In occipital view, the supraoccipital is wide and plate-like, and largely flat, with only a low sagittal ridge extending along the midline (Fig. 5). This sagittal ridge is to varying degrees present in many other early archosauromorphs including *Mesosuchus browni*, *Prolacerta broomi*, *Tanystropheus longobardicus* and *Euparkeria capensis*, but is not apparent in *Garjainia prima*^7,36,46,53,55^.

#### Opisthotic

The opisthotic consists of a somewhat dorsally but largely laterally extended paroccipital process, a broad surface articulating with the supraoccipital dorso-medially and the prootic anteriorly, and a ventral ramus, which extends anteriorly along the exoccipital (Fig. 5). The paroccipital process likely connected with the edge of the posterior parietal flange and the squamosal (Fig. 5). The dorso-medial surface has a shallow V-shaped indentation for the capsulation of the semicircular canals of the inner ear (Fig. 6b). The ventral ramus is quadrangular in shape in posterior view and displaying a distinct ventral projection. Medially it is concave, which is to our knowledge unique for this taxon among non-archosauriform Archosauromorpha. The concavity is likely the articulation point of the opisthotic and exoccipital (Fig. 6b). It is possible that via this formation the exoccipital and opisthotic form the metotic fissure or the hypoglossal foramen, this is however uncertain because the relevant elements are disarticulated. Distally projecting ventral rami are not uncommon among early Archosauromorpha as they are present for example in *Euparkeria capensis* and *Mesosuchus browni*, whereby they are fused to the rest of the braincase, but are distinguishable because of their position and because they border the metotic fissure and foramen ovale^46,55^. In *Prolacerta broomi* and *Pamelaria dolichotrachela* the element is distinctly club-shaped and extends as far as the basal tubera of the basioccipital^49,53^.

#### Prootic

The prootic is a large sheet-like bone which articulates with the opisthotic posteriorly, the supraoccipital postero-dorsally and with the parabasisphenoid antero-ventrally (Figs. 5, 6b). In lateral view, the ampulla and ascending loop of the anterior semicircular canal are visible as a bulging ridge on its anterior side (Figs. 5b, 6b). The crista prootica extends ventrally and it continues into the anterior inferior process, which contacts the clinoid process of the parabasisphenoid. The prootic in PIMUZ T 2477 does not contact the parabasisphenoid posterior to the clinoid process, indicating that the ventral part of the braincase was not fully ossified. In this regard, the braincase is more similar to *Youngina capensis* than to other early archosauromorphs such as *Euparkeria capensis*, *Mesosuchus browni* and *Prolacerta broomi*^46,53,55,56^. The crista prootica and the ampulla for the anterior semicircular canal form the edges of a foramen, likely the opening for the passage of CN-VII (Figs. 5b, 6b). There is some discussion whether this foramen is present in *Prolacerta broomi*^53,60^. The foramen lies ventral to the crista prootica in *Euparkeria capensis* and other Archosauriformes^55,57^. In medial view, two large ridges are visible in a more or less antero-posteriorly oriented plane; these are almost connected by a third curved ridge starting from the anterior end of the dorsal of the large two. This third ridge is less thick and forms the medial edge of a foramen, which is possibly part of the semicircular canal passages. The larger two ridges frame a sack-like cavity with an antero-ventral facing indentation, the main part of the inner ear capsule. The prootic thereby contributes most to the inner ear capsule. This could be the case in other non-archosauriform archosauromorphs as well, but is difficult to discern since in most taxa the opisthotic and prootic have fused into a single structure^7,36,46,55^. The supraoccipital and opisthotic also contribute to the otic capsule (Fig. 6b). No ossified laterosphenoid appears to have been present anterior to the prootic (Figs. 5b, 6b, and supplementary material). The laterosphenoid is considered to be a character of archosauriforms^61^, but has also been described for the non-archosauriform archosauromorph *Azendohsaurus madagaskarensis*^34^. Furthermore, although this element has not been identified, some morphological correlates could hint that the laterosphenoid was ossified in *Mesosuchus browni*^46^. Due to the disarticulated preservation of the braincase of PIMUZ T 2477, the connection between opisthotic and prootic is not fully clear. Therefore, we cannot comment on the position of the foramen ovale.

#### Epipterygoid

An elongate rod-like element is present underneath the basioccipital and parabasisphenoid in PIMUZ T 2477. The element has a ventral structure, which is comprised of two round bosses, whereas dorsally the element is elongated and rod-like. There is a flange or surface projecting posteriorly on the onset of the rod. We hypothesize this element to be the epipterygoid (Figs. 5b, 6b). Similar structures have been identified as epipterygoids in *Mesosuchus browni*^46^ and *Garjainia prima* and the epipterygoid is preserved in articulation in *Proterosuchus fergusi*^46,36,62^. In *Mesosuchus browni* only the rod structure can be seen clearly^46^. The morphology in *Garjainia prima* is very similar as it has two round bosses ventrally and a rodlike structure dorsally^36^. The gap between the two bosses in *Garjainia prima* is much larger than in *M. bassanii* and in *Garjainia prima* the posterior boss is larger than the anterior boss, the taxon does lack the flange of *M. bassanii*^36^. A posterior directed flange (or posterior process) is present in extant Squamata such as *Trachylepis* spp*.* and *Pseudopus apodus*, but this feature could be convergent as it is not apparent in other Squamata^33,52,63^. The rod-like structure is directed anterodorsally in both *Garjainia prima and Mesosuchus browni,* but is directed slightly postero-dorsally in *Proterosuchus fergusi*^36,46,62^. *Proterosuchus fergusi* lacks the ventral bosses, instead the ventral portion of its epipterygoid is expanded with a distinct anterior lamina^62^. In the reconstruction of PIMUZ T 2477, the epipterygoid is positioned onto the pterygoid with the bosses as footplate. The exact location of articulation is difficult to discern as there is no clear facet on the pterygoid (Fig. 5b). The facet for the epipterygoid is also notably absent in *Garjainia prima*^36^. We infer the position based on the following aspects: (1) in this constellation, the rod-like part of the element extends dorsally along the anterior part of the prootic as in all extant Squamata (e.g.^33^); (2) the position of articulation with the pterygoid is often posterior to the articulation with the parabasisphenoid in extant reptiles, and was similarly reconstructed for the early diapsid *Orovenator mayorum*, to which the general morphology of the epipterygoid of *M. bassanii* is also similar^33,64^. In our reconstruction the rod of the epipterygoid is directed postero-dorsally along the prootic, not antero-dorsally as in *Garjainia prima and Mesosuchus browni.* We have made this interpretation because *M. bassanii* lacks an ossified laterosphenoid and can therefore not contact an element antero-dorsally, which is present in the more derived *Garjainia prima*, *Proterosuchus fergusi* and possibly *Mesosuchus browni*, although *Proterosuchus fergusi* does not have an anterior directed epipterygoid despite it having an ossified laterosphenoid^36,46,62^.

### Palatal complex

#### Vomer

The vomer is the anterior-most element in the palatal complex. It is an elongated element and covered most of the palate anteriorly, resulting in very slender choanae laterally (Fig. 3b). The right vomer is better preserved than the left vomer and is still in articulation with the right palatine. Both vomers appear to bear a single file of more or less conical teeth on their ventral side (Fig. 3b). The teeth are positioned antero-posteriorly and are located on the medio-ventral side of the element. However, because a second shorter and laterally situated row of teeth has been identified in other specimens of *Macrocnemus* (PIMUZ T 2476 = ‘Besano II’ specimen and PIMUZ T 2475 = ‘Valporina 1933’ specimen:^3^; T 2474:^8^; T 1559:^12^), and individual teeth were difficult to discern in the synchrotron µCT data, we cannot exclude the presence of a lateral row in PIMUZ T 2477. No septomaxilla has been identified in the CT data, and based on the narrow end of the snout and nature of the articulation between the vomer and premaxilla, it is either absent or underdeveloped as in *Prolacerta broomi*^37^.

#### Palatine

Both palatines are incomplete and therefore their exact shape cannot be determined (Fig. 3b,c) based on the synchrotron µCT data. However, this element has been described in detail for *M.* aff. *fuyuanensis* PIMUZ T 1559 by Jaquier et al.^12^. The right palatine is preserved in articulation with the vomer, whereas the left palatine has disarticulated and is less complete. Like the pterygoid and the vomer, the palatine bears teeth. More anteriorly, the teeth are ordered in two rows that extend diagonally over the element, whereby both tooth rows extend postero-medially. These teeth are conical and bulbous. More posteriorly, the teeth are narrower and the tooth row is projected medially (Fig. 3c). Heterodonty on the palatine and pterygoid of *M. bassanii* has been suggested previously by Kuhn-Schnyder, but he unfortunately did not disclose photos or interpretative drawings^8^. Palatal teeth are known in *Prolacerta broomi*, *Euparkeria capensis* and *Azendohsaurus madagaskarensis*, but are absent in *Eryhthrosuchus africanus* and possibly intraspecifically variably present in *Garjainia prima*^34–37,65^. In *Tanystropheus longobardicus*, they are present in small-sized specimens, but absent in large-sized specimens, which might indicate a taxonomic distinction between these two morphotypes^16^. Like *M. bassanii*, *Prolacerta broomi* has medially projected teeth posteriorly on the element and teeth on the ventral side more anteriorly, although these anterior teeth are described to be ordered in a single file row^60^.

#### Pterygoid

The pterygoids are the largest elements in the palatal complex. They are elongated elements, with anteriorly tapering rami. The pterygoids articulate dorsally with the basipterygoid processes of the parabasisphenoid, laterally with the ectopterygoids, and antero-laterally with the palatines (Fig. 3b). The pterygoids have a distinct concave surface medially for the articulation with the basipterygoid processes on the parabasisphenoid (Fig. 3). On the dorsal side, there is no clear facet for the epipterygoid. In *M. fuyuanensis* PIMUZ T 1559 a facet on the pterygoid for the epipterygoid has tentatively been described^12^. Posterior to the parabasisphenoid facet the quadrate ramus of the pterygoid is located. In the left pterygoid, this ramus is projecting more postero-laterally and is longer, likely due to preservational effects. The lateral projecting transverse process is well developed. The transverse process is flattened medially and thickens more laterally. On its palatal surface, the pterygoid bears two separate forms of teeth. Posteriorly on the element, the teeth are more conical and blunter. They are present along the midline of the element. Anteriorly, the teeth are sharper and narrow and appear on the medial side of the element, sometimes even pointing directly medially, as in *Prolacerta broomi* and proterosuchids^44,66,67^. As for the palatine, only the pterygoids of the small-sized specimens of *Tanystropheus longobardicus* bear teeth on their anterior ramus^16^. It is difficult to determine how many single rows of teeth there are, due to overlapping other bones, but the teeth seem to align in an anterio-posterior direction and not branch out (antero)laterally. There are no teeth observed on the transverse processes, but similar to the vomers, identification of teeth is difficult here as it appears to be obscured by other skull bones in the μCT scan. Peyer described the pterygoid and the vomers of *M. bassanii* in specimen PIMUZ T 2475 and T 2476 and Kuhn-Schnyder in PIMUZ T 2472^3,8^. Their descriptions were based mostly on X-ray images, as these bones are not or incompletely visible at the surface on these specimens. Both authors indicate rows of teeth on the palatal ramus. Kuhn-Schnyder also comments that the posterior teeth look stronger and Peyer indicated an additional row on the transverse processes^3,8^. Pterygoids are also visible in the holotype of *M. fuyuanensis* IVPP V15001 and referred specimen PIMUZ T 1559^12,13^. Medially directed teeth in PIMUZ T 2477 and PIMUZ T 1559 appear to be a preservational artefact as the pterygoid margins are not completely preserved in these specimens. In contrast the holotype of *M. fuyuanensis* lacks medially directed teeth, but clearly has teeth on its transverse process [TMS and SNFS pers. observ.]. Differences in pterygoid shape between *M. fuyuanensis* IVPP V15001 and *M. bassanii* PIMUZ T 2477 are interpreted to reflect preservational differences.

#### Ectopterygoid

The ectopterygoid is a flattened semilunar-shaped bone in ventral view. The ectopterygoid hypothesized for PIMUZ T 1559^12^ does not resemble the morphology we observe in the synchrotron µCT data for PIMUZ T 2477, which has both ectopterygoids well preserved. The ectopterygoid was also described as a curved structure by Kuhn-Schnyder^8^, which he interpreted to be the ectopterygoid, although he provided no figures to display his observation^8^. We deem our interpretation more likely than the identification in PIMUZ T 1559^12^. The articulation with the pterygoid is hard to establish since no clear articulation surfaces are present. The disassociation of the ectopterygoids and pterygoids indicates they were not in a tight connection. The jugal and pterygoid facets of the ectopterygoid are roughly the same size, it thereby lacks a distinct head of the jugal facet as in *Azendohsaurus madagaskarensis*^34^. The position of the ectopterygoid in *M. bassanii* was inferred from comparisons with *Prolacerta broomi*^37^ and modern Lepidosauria (e.g.^33,63^) (Fig. 3), it also resembles the proposed position of the ectopterygoid in *Azendohsaurus madagaskarensis*^34^. The ectopterygoid bears no teeth. This was to be expected as ectopterygoidal teeth are virtually absent among archosauromorphs, but do occur in non-saurian diapsids^68^.

### Lower jaw

#### Dentary

**Figure 7:**
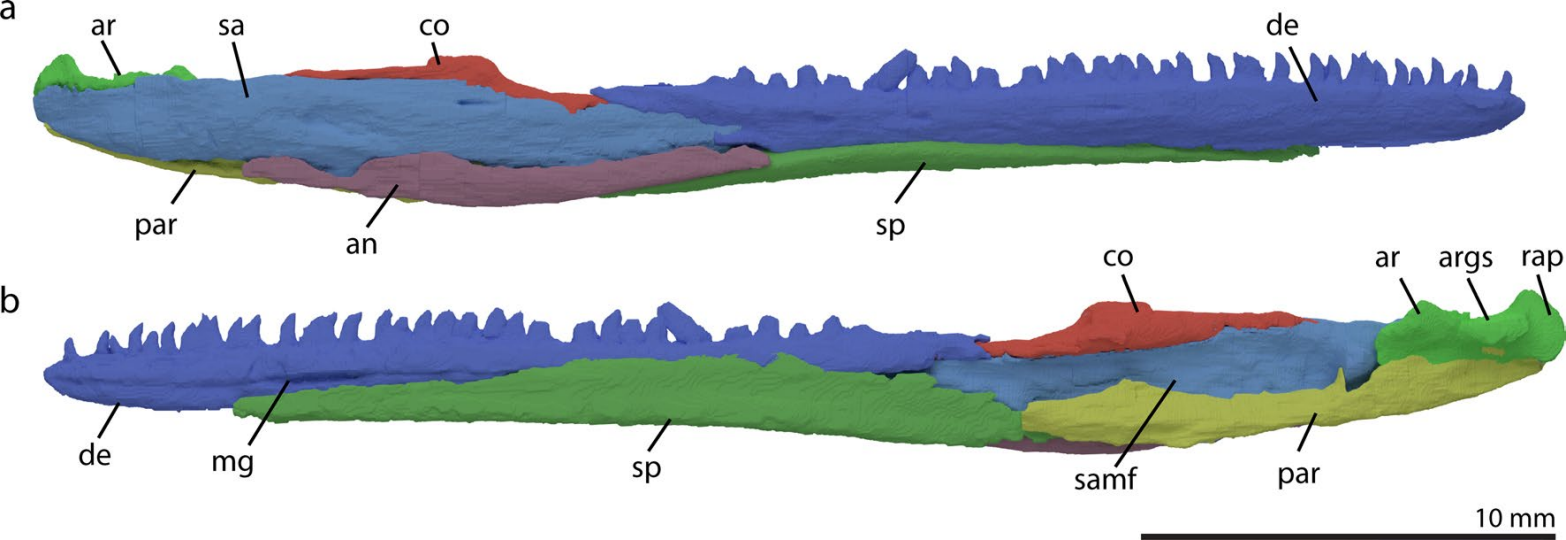
Digital rendering of the reconstructed right mandible of *M. bassanii* specimen PIMUZ T 2477 in lateral view (**a**) and medial (**b**) view. *an* angular, *ar* articular, *args* articular glenoid surface, *co* coronoid, *de* dentary, *mg* Meckelian groove, *par* prearticular, *rap* retroarticular process, *sa* surangular, *samf* surangular adductor muscle fossa, *sp* splenial.

The dentary is the anterior-most element in the hemimandible. It articulates with the splenial along most of its ventro-medial side, and articulates with the surangular and coronoid postero-dorsally and with the angular postero-ventrally (Figs. 1, 7). It is the largest element in the hemimandible, as its length represents around two thirds of the entire hemimandible. It is also the only tooth-bearing element in the lower jaw. On its anterior half, the dorsal margin is slightly curved ventrally. Like the premaxillae, the dentaries do not form a fully formed symphyseal structure, but rather touch on the midline and both medial tips are smooth in texture. This morphology fits in symphyseal class I, similar to most other early archosauromorphs such as *Prolacerta broomi*, *Mesosuchus browni*, *Proterosuchus fergusi* and *Euparkeria capensis*^69^. Small pits can be seen aligned antero-posteriorly along the lateral side of the dentary, possibly representing sensory pits. These pits are also present in *Prolacerta broomi* and *Tanystropheus longobardicus*^37,43^. The dentary has around 35 tooth positions (Fig. 7). The teeth are slightly re-curved, not labiolingually compressed, similar to the premaxillary and maxillary teeth. The dentition terminates distinctly anterior to the posterior extent of the dentition of the maxilla (Fig. 1). The dentary terminates in two processes posteriorly, a long and tapering postero-dorsal process and a very short postero-ventral process (Fig. 7a). Antero-posteriorly along the medial surface of the dentary, a thin groove for the Meckelian cartilage is located shortly ventral to the mid-height of the bone (Fig. 7b). There has been some discussion on the differing morphology of *M. bassanii* and *M. fuyuanensis*. On the second described specimen of *M. fuyuanensis*, GMPKU-P-3001, the authors suggested that the dentary was forked posteriorly over the surangular and angular, excluding the splenial from lateral exposure^14^, which was later disputed^12^. The specimen described here, PIMUZ T 2477, has a similar morphology to what has been described for other *M. bassanii* specimens such as PIMUZ T 4822, whereby the splenial forms the ventral portion of the anterior hemimandible in lateral view, not a ventral fork of the dentary^12^. All other specimens of *Macrocnemus* show similar morphology to what is described for PIMUZ T 2477, including the holotype of *M. fuyuanensis* [TMS and SNF pers. observ.].

#### Splenial

The splenial lies along most of the ventro-medial side of the dentary and connects to the prearticular, angular, and surangular posteriorly. It is an elongate structure with a U-shape in anterior or posterior view as it wraps around the dentary ventrally, exposing part of its ventral side laterally (Fig. 7). The splenial widens in medial view to about the last tooth positions in the dentary, and then tapers again into a long postero-ventral process that articulates ventrally with the prearticular (Fig. 7b). The splenial does not reach the mandibular symphysis.

#### Angular

The angular forms a portion of the lateral surface of the posterior hemimandible. It is an elongated bowed structure with a dorsally concave and ventrally convex margin, positioned between the surangular dorsally, the splenial ventrally, the dentary antero-dorsally, and the prearticular postero-ventrally (Fig. 7). This morphology leads to a convex suture between the angular and surangular, similar to for example *Prolacerta broomi* and *Mesosuchus browni*, but contrary to *Garjainia prima*^11,36,37^.

#### Surangular

The surangular constitutes the largest part of the posterior hemimandible and forms the lateral margin of the posterior surface (Fig. 7). It articulates with the dentary anteriorly, the coronoid dorsally, the angular ventrally, the prearticular postero-ventrally and the articular postero-medially. Medially, the surangular is concave as it frames the adductor fossa laterally (Fig. 7b).

#### Coronoid

Dorsal to the surangular a coronoid bone is present. The dorsal inclination of the bone in both anterior and posterior direction is very shallow, giving the element a very wide angled triangular outline. The element contains a small, rounded coronoid process on its dorsal margin. The contact between the surangular and coronoid is very tightly sutured, with a flat contact in PIMUZ T 2477, whereby it almost seems to be only a process of the coronoid itself (Fig. 7). The presence of a coronoid bone in *Tanystropheus longobardicus* is currently unclear^43^. In the tanystropheid *Langobardisaurus pandolfii* a very tall coronoid process is present, but because the mandibular sutures cannot be discerned confidently, it is unclear whether it is formed by the surangular (potentially fused with the coronoid bone) or a separate coronoid element^70^.

#### Prearticular

The prearticular is an elongated element, located posteriorly on the medial side of the hemimandible. It encloses the articular dorso-laterally and contacts the surangular ventrally, and the splenial anteriorly (Fig. 7). Because the posterior extent of the angular is not entirely clear, it cannot be determined unambiguously whether the prearticular was exposed in lateral view (Fig. 7a). The prearticular does not reach the dorso-medial margin of the hemimandible allowing the adductor fossa to be largely exposed medially. This is a common morphology among early archosauromorphs, but is notably different in *Trilophosaurus buettneri*^40^.

#### Articular

The articular is the posterior-most element in the hemimandible. The glenoid fossa which forms the articulation facet for the quadrate is located on the dorsal surface of the articular and cup-shaped (Fig. 7). The lateral wall of the glenoid fossa is higher than the medial wall; however, the medial wall is thicker. The articular has a stout but short upturned retro-articular process, posterior to this facet, similar to that in *Prolacerta broomi, Mesosuchus browni* and to a lesser degree in *Protorosaurus speneri*^11,37,38^. The process is keel-shaped. It likewise has a thickened surface anterior to the articulation facet. The medial margin of the glenoid fossa has a short medially directed extension. The articular is positioned between the surangular antero-laterally and the prearticular ventrally (Fig. 7).

### Atlas-axis complex

**Figure 8:**
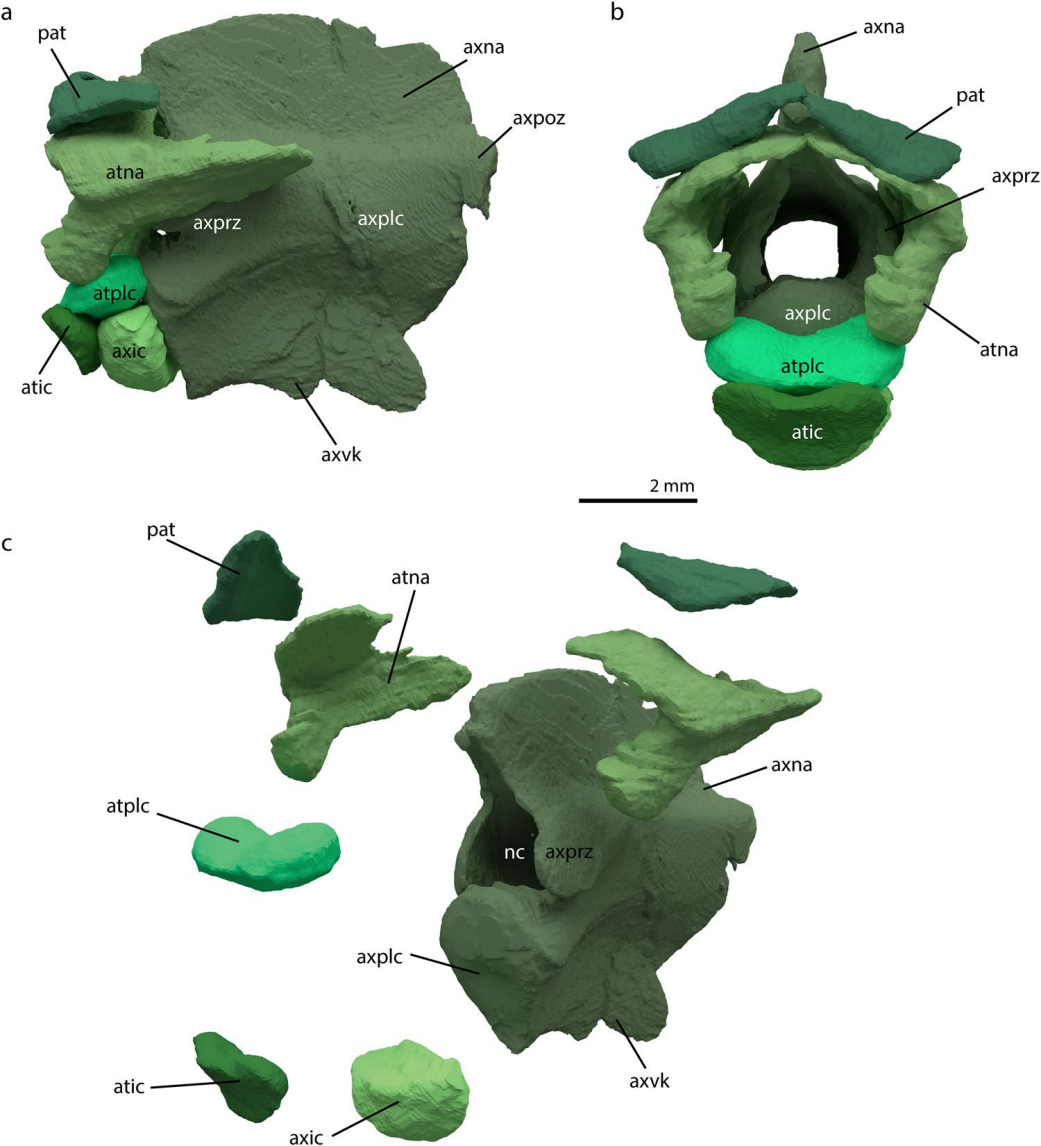
Digital rendering of the reconstructed atlas-axis complex of *M. bassanii* specimen PIMUZ T 2477 in left lateral view (**a**), anterior view (**b**) and separated elements in antero-lateral view (**c**). *atic* atlas intercentrum, *atna* atlas neural arch, *atplc* atlas pleurocentrum, *axic* axis intercentrum, *axna* axis neural arch, *axplc* axis pleurocentrum, *axpoz* axis postzygapophysis, *axprz* axis prezygapophysis, *axvk* axis ventral keel, *nc* neural canal, *pat* proatlas.

The atlas-axis complex consists of eight elements: the axis, axis intercentrum, atlas pleurocentrum, atlas intercentrum, two atlantal neural arches and two pro-atlases (Fig. 8). Most prominent among these is the axis (excluding the intercentrum) or epistropheus. It has a prominent neural arch, the neural spine of which projects dorsally with a slight curvature anteriorly, such a curvature is likewise present in *Augustaburiania vatagini* and *Tanystropheus longobardicus*^7,71^. The zygapophyses protrude anteriorly and posteriorly on the lateral sides of the neural arch (Fig. 8). A large keel is present on the ventral side of the centrum. A very large opening for the passage of the neural canal is visible in anterior view between the zygapophyses. The axis has a large cavity throughout its central body, through which the central nerve cord presumably passes as has been described for the postaxial cervical vertebrae of *Tanystropheus longobardicus*^72^. The axis is incomplete posteriorly and misses its posterior section. Furthermore, a dorsoventrally oriented crack runs through the neural spine, neural arch, and pleurocentrum of the axis. Part of the ventral keel is broken as a result of this crack, and it therefore appears that the ventral keel projects posteriorly. However, comparison with the complete axis in the *M. bassanii* specimen PIMUZ T4822^12^ reveals that this is in fact a preservational artifact. The axis intercentrum is a round element contacting the anterior facet of the axis pleurocentrum posteriorly, antero-dorsally to its ventral keel. The axis intercentrum has a flattened dorsal surface on which the atlas pleurocentrum is likely positioned. Anteriorly it is contacted by the atlas intercentrum.

The atlas pleurocentrum is a crescent-shaped bulbous structure (Fig. 8). It is contacted on both lateral sides by the atlas neural arches. Dorsally the element is concave, either to facilitate the articulation with the basioccipital condyle or to form the floor of the neural canal. Like the atlas pleurocentrum, the atlas intercentrum is a crescent-shaped element (Fig. 8). It is the most slender of the atlas-axis components in the dorsoventral plane. Posteriorly it contacts the axis intercentrum and atlas pleurocentrum. Its dorsal-most margin could contribute to the contact between the basioccipital and atlas pleurocentrum. The atlas neural arches are not fused to the pleuro- or intercentra (Fig. 8). They are paired, triradial structures. The posterior flange of the atlantal neural arch lies on the prezygapophysis of the axis. It is medially convex and fits onto the projection formed by the prezygapophysis. The anterior part of the atlantal neural arch consists of two flanges. Of these, the more bulbous one faces antero-ventrally and articulates with the atlas pleurocentrum. The more flattened flange projects dorso-medially and possibly touches the same structure of the opposing atlas neural arch at its distal margin (Fig. 8). In this manner, both neural arches create an arched roof around the neural canal. The paired pro-atlases lie dorsally of the antero-medial flanges of the atlantal neural arches. They are flat triangular structures. They potentially articulate with the dorsal side of the axis, but this cannot be determined unambiguously because these elements are disarticulated in PIMUZ T 2477.

The axis (excluding intercentrum) is the only element of the atlas-axis complex that has been unambiguously recognized in *Macrocnemus* in earlier studies (e.g.^3,12,17^). Atlantal neural arches have been recognized as atlas structures in the past, but have been described as atlas^17^ or atlantal rib^14^. Unsurprisingly, the axis is very similar to that of the tanystropheids *Augustaburiania vatagini, Amotosaurus rotfeldensis* and *Tanystropheus longobardicus* in the presence of an antero-dorsally sloped neural spine^7,71,73^. Atlantal neural arches have so far only been identified for *Tanystropheus longobardicus* among tanystropheids^7^. Among non-archosauriform archosauromorphs, the atlas-axis complex has been described in detail for the allokotosaurs *Azendohsaurus madagaskarensis*, *Trilophosaurus buettneri*, and *Pamelaria dolichotrachela*^40,49,51^. In the former two taxa, the atlas pleurocentrum and axis intercentrum are fused, but the overall morphology of all individual elements is otherwise largely similar to that of PIMUZ T 2477*.* However, the atlas neural arches of *Azendohsaurus madagaskarensis* contact the atlas intercentrum, whereas in *Macrocnemus* they only contact the atlas pleurocentrum^51^. The axes of *Azendohsaurus madagaskarensis* and *Trilophosaurus buettneri* are likewise similar to *M. bassanii* in having a pronounced neural arch and prezygapophyses closely fused to the central body. However, these taxa lack a pronounced axial ventral keel as seen in *M. bassanii*^40,51^. In *Pamelaria dolichotrachela* the atlas-axis complex consists of triradiate atlantal neural arches articulating to the prezygapophyses of the axis, which is similar in morphology to PIMUZ T 2477. However, the presumed atlas pleurocentrum is described as being quadrangular in anterior view with distinct processes for the atlantal neural arches, whereas PIMUZ T 2477 has a more rounded atlas pleurocentrum. Unfortunately, these elements were not figured in the description, which hampers a detailed comparison to *Macrocnemus*^49^.

## Discussion

### Cranial kinesis

There has been much debate over the motility of the quadrate and posterior palate of *M. bassanii*^8,39^. An earlier interpretation suggested that the cranium must have been streptostylic due to the connection of the squamosal and quadrate in the deeply concave facet of the squamosal^8^. Later studies agreed that this articulation would give room for movement^17^. However, other authors concluded that this could not have been the case due to the connection of the quadrate with the pterygoid^39^. They hypothesized that, due to the large basipterygoid processes the posterior region of the palate was movable, rendering similar independent movement of the quadrate very improbable. They likewise argued that the squamosal-quadrate joint is more rigid than previously thought^39^. According to our reconstruction, we infer the articulation between the squamosal and quadrate to be very tight in PIMUZ T 2477. Anterior movement of the quadrate head cannot be completely excluded, but would have been very limited at most. Moreover, the postero-ventral process of the squamosal extends over the dorsal head of the quadrate, thereby inhibiting posteriorly directed movement of that head. The likely overlapping contact between the pterygoid and ectopterygoid could be an indication that some small movement was possible along this suture, but this cannot be stated confidently. We therefore tentatively infer that there was little to no streptostyly present in the skull of *M. bassanii*.

### Potential phylogenetic information

The high resolution synchrotron μCT data of PIMUZ T 2477 allow for a refined three-dimensional virtual reconstruction of the skull of *Macrocnemus bassanii* in high detail. Previous tooth count estimations of the marginal dentition could be confirmed. We observed new details which are potentially valuable in phylogenetic analyses; among which are: The morphology of the processes of the premaxilla, the shape of the retroarticular process and the morphology of the ventral ramus of the opisthotic. Although they are less well preserved we tentatively observe new information on the dental allignment and morphology of the palatal elements, especially the palatine. Although further studies are needed in this regard. We further confirm the presence and describe the shape of the epipterygoid, ectopterygoid and quadratojugal in *M. bassanii*, as well as the rod-like anterior morphology of the nasals, whereas the posterior parts could not be reconstructed in PIMUZ T 2477. The 3D model adds to the growing data base of archosauromorph reptiles, that will allow further comparative approaches such as biomechanical studies for the group in the future. Our study highlights the potential for the possibility of 3D reconstructions of specimens that have been preserved in 2D, for example in shale strata, and their added worth in describing morphological characters.

## Supplementary information

Supplementary Information 1.

Supplementary Information 2.

Supplementary Information 3.

Supplementary Information 4.

Supplementary Information 5.
