# Supplementary figures and images for: Cranial morphology of the tanystropheid Macrocnemus bassanii unveiled using synchrotron microtomography
Source: Sci Rep. 2020 Jul 24;10:12412. doi: 10.1038/s41598-020-68912-4 (PMC7381672; doi:10.1038/s41598-020-68912-4)

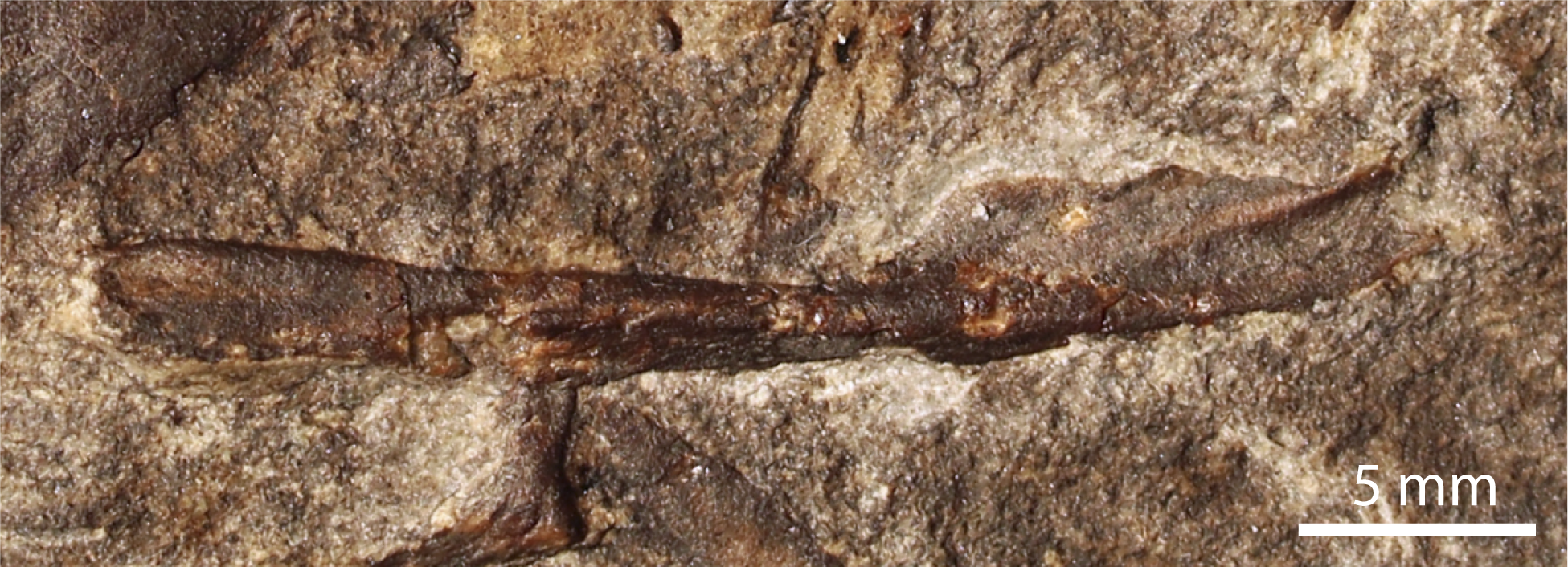

Supplement: Supplementary file 1 — Supplementary Information 1. [file 41598_2020_68912_MOESM1_ESM.tif]
