# Supplementary material for: Cranial morphology of the tanystropheid Macrocnemus bassanii unveiled using synchrotron microtomography
Source: Sci Rep. 2020 Jul 24;10:12412. doi: 10.1038/s41598-020-68912-4 (PMC7381672; doi:10.1038/s41598-020-68912-4)
Supplement: Supplementary file 5 — Supplementary Information 5. [file 41598_2020_68912_MOESM5_ESM.pdf]

Table S1: Observed and segmented cranial elements and their usage in rendering the model of PIMUZ T 2477.

Element	Preservation/observation	Model images
premaxilla	Left and right	Both used
maxilla	Left and right	Left used and mirrored to right
nasal	Premaxillary process? L/R?	Used and mirrored
dentary	Left and right	Left used and mirrored to right
splenial	Left and right	Left used and mirrored to right
coronoid	Left and right	Right used and mirrored to left
angular	Left	Left used and mirrored to right
surangular	Left	Left used and mirrored to right
prearticular	Left	Left used and mirrored to right
articular	Left	Left used and mirrored to right
jugal	Left and right	Left used and mirrored to right
prefrontal	Left and right	Left used and mirrored to right
frontal	Left and right	Both used
postfrontal	Left	Left used and mirrored to right
postorbital	Left and right	Left used and mirrored to right
squamosal	Left	Left used and mirrored to right
parietal	Left and right	Both used
quadrate	Left	Left used and mirrored to right
quadratojugal?	Left	Left used and mirrored to right
epipterygoid?	Left?	Used and mirrored
pterygoid	Left and right	Right used and mirrored to left
ectopterygoid	Left and right	Both used
palatine	Left and right	Both used
vomer	Left and right	Both used
basioccipital	present	used
parabasisphenoid	present	used
exoccipital	Left and right	Right used and mirrored to left
opistothic	Left and right	Right used and mirrored to left
prootic	Left and right	Right used and mirrored to left
supraoccipital	present	used
Axis pleurocentrum	present	used
Axis intercentrum	present	used
Atlas pleurocentrum	present	used
Atlas intercentrum	present	used
Atlas neural arch	Left and right	Left used and mirrored to right
proatlas	Left and right	Both used
